# Safety and tolerability of 6-month supplementation with a vitamin D, calcium and leucine-enriched whey protein medical nutrition drink in sarcopenic older adults

**DOI:** 10.1007/s40520-020-01519-x

**Published:** 2020-03-12

**Authors:** Jürgen M. Bauer, Lucia Mikušová, Sjors Verlaan, Ivan Bautmans, Kirsten Brandt, Lorenzo M. Donini, Marcello Maggio, Tony Mets, Sander L. J. Wijers, Jossie A. Garthoff, Yvette Luiking, Cornel Sieber, Tommy Cederholm, the PROVIDE Consortium, Jürgen M. Bauer, Sjors Verlaan, Ivan Bautmans, Kirsten Brandt, Lorenzo M. Donini, Marcello Maggio, Marion E. T. McMurdo, Tony Mets, Chris Seal, Sander L. J. Wijers, Gian Paolo Ceda, Giuseppe De Vito, Gilbert Donders, Michael Drey, Carolyn Greig, Ulf Holmbäck, Marco Narici, Jamie McPhee, Eleonora Poggiogalle, Dermot Power, Aldo Scafoglieri, Ralf Schultz, Cornel Sieber, Tommy Cederholm

**Affiliations:** 1 Center for Geriatric Medicine, University Heidelberg, Agaplesion Bethanien Krankenhaus Heidelberg, Heidelberg, Germany; 2 Danone Nutricia Research, Nutricia Advanced Medical Nutrition, Utrecht, The Netherlands; 3 Department of Internal Medicine, Section of Gerontology and Geriatrics, VU, Amsterdam University Medical Center, Amsterdam, The Netherlands; 4 Frailty in Ageing Research Group (FRIA), Vrije Universiteit Brussel (VUB), Brussels, Belgium; 5 Human Nutrition Research Centre, School of Agriculture, Food and Rural Development, Newcastle University Institute for Ageing, Newcastle University, Newcastle upon Tyne, UK; 6 Department of Experimental Medicine, Section of Medical Pathophysiology, Endocrinology and Human Nutrition, “Sapienza” University of Rome, Rome, Italy; 7 Department of Clinical and Experimental Medicine, Section of Geriatrics, University of Parma, Parma, Italy; 8 Danone Food Safety Center, Utrecht, The Netherlands; 9 Friedrich-Alexander-Universität Erlangen-Nürnberg, Erlangen, Germany; 10 Department of Public Health and Caring Sciences/Clinical Nutrition and Metabolism, Department of Geriatric Medicine, Uppsala University Hospital, Uppsala, Sweden

**Keywords:** Nutrition, Safety, Tolerability, Sarcopenia, Whey protein, Vitamin D

## Abstract

**Aims:**

Safety and tolerability of prolonged supplementation with a vitamin D, calcium and leucine-enriched whey protein medical nutrition drink (WP-MND) was evaluated in sarcopenic older adults.

**Methods:**

A 13-week double-blinded, randomized, isocaloric placebo-controlled trial (PROVIDE study; *n* = 380) was extended with a voluntary 13-week open-label extension (OLE). OLE participants were randomized to receive daily 1 or 2 servings of WP-MND (21 g protein, 3 g leucine, 10 µg vitD and 500 mg calcium per serving). Gastro-intestinal tolerability, kidney function and serum levels of calcidiol, parathyroid hormone (PTH) and calcium were evaluated at week 0, 13 and 26.

**Results and discussion:**

In response to the high daily protein intake (median1.5; IQR: 1.3, 1.7 g/kg BW/day), the estimated glomerular filtration rate (eGFR) increased in the test group during the RCT (*p* = 0.013). The same trend was observed for those participants with moderate chronic kidney disease. During OLE no eGFR change was observed in any of the groups. Serum calcidiol and calcium reached a plateau after 13-week WP-MND supplementation. As expected, PTH significantly changed in the opposite direction, decreasing during RCT in the test group (T vs C: *p* < 0.001) and during OLE in former control groups. During RCT, 20/366 participants with normal baseline calcidiol reached levels ≥ 100 nmol/L (T: *n* = 18; C: *n* = 2) and 6 developed albumin-corrected calcium levels > 2.55 mmol/L (T: *n* = 3; C: *n* = 3), without associated adverse events.

**Conclusion:**

A 6 months intervention with up to 2 servings of WP-MND did neither result in kidney function deterioration nor symptoms of vitamin D or calcium toxicity. The product was overall well tolerated.

## Introduction

Sufficient dietary intake of protein, and an adequate vitamin D and calcium status has been proposed as a way to attenuate age-related loss of muscle and bone [1–8]. This is especially important for frail, sarcopenic and osteoporotic older adults who are at risk of falls, fractures and subsequent hospitalization or institutionalization [9–11]. The European Society for Clinical Nutrition and Metabolism (ESPEN), the European Union Geriatric Medicine Society (EUGMS), and the European Society for Clinical and Economic Aspects of Osteoporosis and Osteoarthritis (ESCEO) recommend a protein intake of 1.0–1.2 g protein/kg bodyweight (BW)/day (d) for healthy older individuals and 1.2–1.5 g/kg BW/day for geriatric patients with acute and/or chronic diseases [7, 9, 12]. The Institute of Medicine in the USA and ESCEO recommend a daily intake of 800 IU (= 20 µg) vitamin D_3_ for people of 71 years and older to sustain 25-hydroxyvitamin D (calcidiol) levels above 50 nmol/L [5, 7, 13–15]. Calcidiol is the precursor of the active, but short-lived 1,25-hydroxyvitamin D (calcitriol), and represents an integrated marker of vitamin D status as a result of endogenous synthesis from sun exposure and dietary intake [5]. The recommended daily allowance (RDA) for calcium for people of 71 years and older is 1200 mg/day [5]. Despite the recommendations above, insufficient intake of protein, vitamin D and calcium is still common in community-dwelling older adults [16, 17]. This may also partly explain the observed 45% prevalence of vitamin D deficiency, i.e. calcidiol < 50 nmol/L, in a large cohort of community-dwelling older (≥ 65 years) individuals [18].

Previously, an intervention with a vitamin D, calcium and leucine-enriched whey protein medical nutrition drink was shown to enhance muscle mass and improve lower-extremity function in sarcopenic older adults [19]. To obtain these beneficial effects, two servings of the medical nutrition drink and thereby high amounts of protein (42 g), leucine (6 g), vitamin D (40 µg) and calcium (1000 mg) per day were consumed on top of the regular diet. However, this may cause safety concerns in this vulnerable older target population with regard to kidney function and potential vitamin D or calcium toxicity. Older adults experience kidney function decline with age [20] and those with moderate or severe chronic kidney disease are advised to restrict protein intake and/or have their kidney function monitored on a regular basis [7, 9, 12]. Vitamin D or calcium toxicity related hypercalcemia may facilitate the formation of kidney stones and calcification of soft and vascular tissues, and result in acute symptoms such as nausea, vomiting, increased thirst and depression [21].

The aim of this post-hoc evaluation was to determine the effect of 6 months supplementation with a vitamin D, calcium and leucine-enriched whey protein medical nutrition drink (WP-MND) on kidney function and on vitamin D, PTH and calcium levels in sarcopenic older adults. For this purpose, we extended the 13-week randomized placebo-controlled trial [19], with a subsequent voluntary 13-week open-label study period with WP-MND supplementation in both the former test and control groups. In addition, general safety was evaluated with parameters of liver function and registration of adverse events and tolerability was evaluated by a questionnaire addressing symptoms of gastrointestinal discomfort.

## Methods

### Participants

As described in detail before [19, 22], participants were recruited from 18 study centers in 6 European countries. Older adults (≥ 65 years) were eligible when they suffered from sarcopenia, defined as low skeletal muscle mass index (SMI) combined with mild to moderate limitations in physical performance (for definitions applied see reference [19]). People with liver or kidney failure were excluded from participation. Known kidney failure was defined in the published protocol as estimated glomerular filtration rate (eGFR) below 30 mL/min/1.73 m^2^, what means that participants with moderate chronic kidney disease (eGFR between 30 and 60) were eligible for participation in the study. For this group, Bauer et al. recommend that they adhere to general protein recommendations for older people (1.0–1.5 g protein/kg BW/day), but have their GFR monitored twice a year [9]. Other exclusion criteria relevant were: (a) intake of a high protein diet 3 months before start of or during the study, (b) intake of a protein- or amino acid- containing nutritional supplement 3 months before start of or during the study, (c) consumption of more than 22 µg/day vitamin D from vitamin supplement use, (d) consumption of 11.25–22 µg vitamin D daily from vitamin supplement use in combination with a serum calcidiol concentration ≥ 50 nmol/L, (e) consumption of more than 500 mg calcium daily from mineral supplement use.

### Study design

The 13-week double-blinded RCT (known as the PROVIDE study) involved consumption of 2 servings of a vitamin D, calcium and leucine-enriched, whey protein medical nutrition drink (WP-MND) in the test group or 2 servings of an iso-caloric drink with only carbohydrates, fat and some trace elements in the control group [19]. This RCT was extended with a 13-week open-label extension (OLE) study period. Participants in the RCT who volunteered to participate in the OLE part of the study, were randomized to receive either 1 or 2 servings of the WP-MND (FortiFit, Nutricia N.V., the Netherlands). One serving of WP-MND contained 21 g total protein, including 20 g whey protein and 3 g total leucine, 9 g carbohydrates, 3 g fat, 800 IU (20 µg) vitamin D_3_, 500 mg calcium, and a mixture of vitamins, minerals and fibers (for detailed composition see online supplementary material—Supplemental Table 1 of Bauer et al. [19]). The one-serving group took the WP-MND before breakfast. In the two-serving group, the regimen was the same as in the intervention group of the RCT, i.e. before breakfast and lunch [19].

### Outcome measures

Parameters were assessed prior to the RCT (baseline RCT), at the end of the RCT, which was also the start of the OLE part of the study (week 13, Baseline OLE), and after the OLE study period (week 26). For determination of blood parameters, approximately 25.5 mL of venous blood was obtained at each visit. Blood samples in which creatinine (µmol/L), calcidiol (nmol/L), parathyroid hormone (PTH, pmol/L), calcium (mmol/L) and albumin (g/L) were determined, were left at room temperature for 30 min and then centrifuged. Aliquots of serum were stored at − 20 or – 80 °C until analysis. These serum samples were analyzed at the Reinier de Graaf Groep medical laboratory, Delft, the Netherlands. Blood samples obtained for determination of the other parameters were processed and determined at the local laboratories on site, according to local procedures.

### Kidney function

Kidney function was defined by the estimated glomerular filtration rate (eGFR, mL/min/1.73 m^2^) that was calculated with serum creatinine (Creat, µmol/L), using the Chronic Kidney Disease Epidemiology (CKD-EPI) Equation as described by Levey et al. [23]. Briefly, the CKD-EPI equation includes log [Creat] (modeled as a 2-slope linear spline with sex-specific 0.7 mg/dL in women and a 0.9 mg/dL in men), and gender, race, and age on a natural scale.

### Vitamin D and calcium metabolism

Analytical testing for total serum calcidiol was performed using chemiluminescense micro-particulate immunoassay (Abbott Laboratories, Wiesbaden, Germany). To determine the effects of changes in the serum calcidiol on vitamin D metabolism, serum PTH and calcium were determined as well. Serum intact PTH concentrations were measured in serum using an ELISA [Intact parathyroid hormone, MD Biosciences Inc., St. Paul, MN 55108], with an intra-assay CV of 3.4%. For albumin-corrected calcium, total serum calcium and albumin were measured by an automated system [Instrumentation Laboratories UK Ltd, Cheshire, UK]. The intra-assay CV for serum calcium and albumin was 1.7% and 1.8%, respectively. Serum calcium (Ca) normally ranges between 2.15 and 2.55 mmol/L [24, 25]; therefore, hypercalcemia was defined as [Ca] > 2.55 mmol/L [25]. Serum calcium was adjusted for albumin according to the equation described by James et al. [26]:

$${\text{Adjusted }}\left[ {{\text{Ca}}} \right] \, = \, \left[ {\text{total Ca}} \right] \, + \, 0.0{2 }* \, \left( {{4}0 - \left[ {{\text{albumin}}} \right]} \right)$$

In participants with calcidiol levels ≥ 100 nmol/L at baseline or during the study, serum calcium levels and adverse effects were closely evaluated.

### General safety and gastrointestinal tolerability

Liver function was assessed at the local laboratories by determination of ALanine AminoTransferase (ALAT, U/L), ASparate AminoTransferase (ASAT, U/L), alkaline phosphatase (U/L), and gamma-Glutamyl Transpeptidase (ɣ-GT, U/L). Number and type of adverse events were registered. An adverse event was defined as any untoward medical occurrence in a clinical trial participant who was administered a study product, not necessarily implying a causal relationship with the intervention. The occurrence of adverse events was also checked between visits with intermittent phone calls. Gastrointestinal (GI) tolerability was assessed using a GI questionnaire at baseline, after 13 weeks and after 26 weeks of intervention. This questionnaire covered a range of GI symptoms: nausea, belching, feeling of fullness, vomiting, abdominal distension, flatulence, diarrhoea, constipation, dry mouth and thirst with a four-point scale (absent/mild /moderate/severe).

### Statistical analysis

All the analysis of the main RCT paper [19] were done on the intention to treat (ITT) data set, while the safety and tolerability evaluation of the current paper was performed on the AST data set (*n* = 379 subjects), which excludes one subject in the control group who was randomized but did not start the intervention.

Normally distributed data were described as mean (SD or range) and analysed using the two-sample and paired *t* test for between and within-group differences, respectively. Not normally distributed data were described as median (IQR or range) and analysed using the Mann–Whitney test for between-group differences and the Wilcoxon Signed-Rank test for within-group changes. A *p *value < 0.05 indicates a significant difference between groups. All statistical analyses were performed using SAS software (version 9.4; SAS, Inc, Cary, NC).

## Results

### *Participants flow *(Fig. 1)

**Fig. 1:**
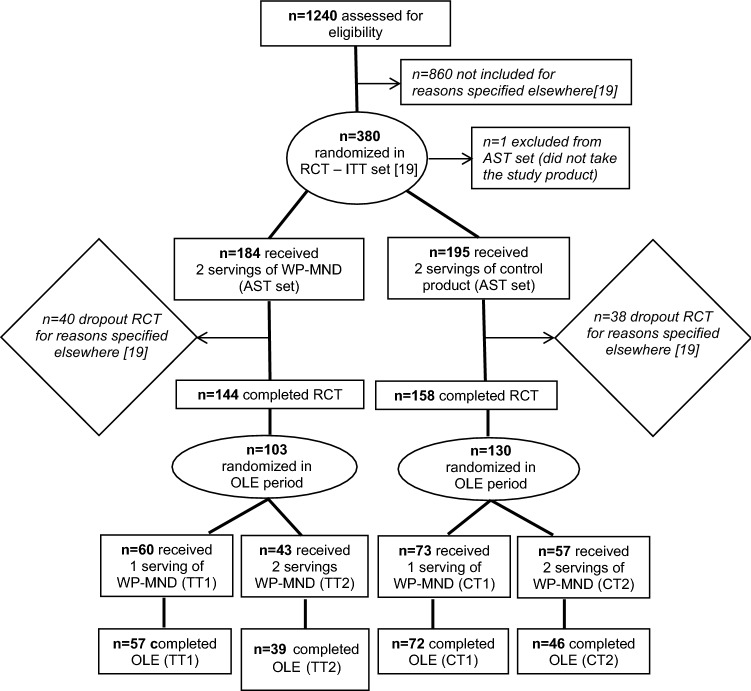
Participants flow. WP-MND, a vitamin D and leucine-enriched whey protein medical nutrition drink; T, test group during RCT; C, Control group during RCT; OLE, open-label extension; TT1, former RCT Test group receiving 1 serving of WP-MND during OLE; TT2, former RCT Test group receiving 2 servings of WP-MND during OLE; CT1, former RCT Control group receiving 1 serving of WP-MND during OLE; CT2, former RCT Control group receiving 2 servings of WP-MND during OLE

At the end of the 13-week RCT, 233 participants in the PROVIDE study agreed to participate in the OLE sequel of the study (test (T): *n* = 103; control (C): *n* = 130). 133 subjects were randomized into the 1-serving groups (CT1 and TT1) and 100 subjects were randomized into the 2-serving groups (CT2 and TT2). At the end of the OLE study, 214 participants completed the study (former T group receiving 1 serving of WP-MND during OLE (TT1): *n* = 57, former T group receiving 2 serving of WP-MND during OLE (TT2): *n* = 39, former C group receiving 1 serving of WP-MND during OLE (CT1): *n* = 72, former C group receiving 2 serving of WP-MND during OLE (CT2): *n* = 46), and 19 participants exited the study before week 26.

### Characteristics of the study population

Baseline characteristics of the RCT population are shown in the paper by Bauer et al. [19] and in Table 1 together with week 13 baseline characteristics of the 4 different OLE groups. Characteristics were not different between groups, neither at baseline for the RCT nor at baseline for the OLE study period, apart from a difference in total protein intake, serum calcidiol and PTH at the start of the OLE study period due to prior intervention in the RCT, which excludes potential selection bias in the OLE extension.

**Table 1:** Baseline (week 0) characteristics of RCT participants (*n* = 279, AST data set) and baseline (week 13) characteristics of participants in open-label extension of the study (OLE) (*n* = 233, AST data set)

	Statistic	Total RCT (*n* = 379)	Week 0 (baseline RCT)			Week 13 (baseline OLE)			
			Control (n = 195)	Test (n = 184)	Total OLE (n = 233)	RCT control (n = 130)		RCT Test (n = 103)	
						CT1 (n = 73)	CT2 (n = 57)	TT1 (n = 60)	TT2 (n = 43)
Age (years)	Mean (SD)	77.7 (6.9)	78.1 (7.0)	77.3 (6.7)	77.4 (6.7)	77.8 (6.7)	78.5 (6.9)	75.6 (6.1)	77.4 (7.2)
Sex (male)	*n* (%)	131 (34.6)	67 (34.4)	64 (34.8)	90 (38.6)	27 (37.0)	25 (43.9)	25 (41.7)	13 (30.2)
Living independently	*n* (%)	328 (86.5)	166 (85.1)	162 (88.0)	209 (89.7)	65 (89.0)	47 (82.5)	58 (96.7)	39 (90.7)
MNA-SF, score	Mean (SD)	13.1 (1.3)	13.1 (1.5)	13.2 (1.2)	13.2 (1.2)	13.2 (1.2)	13.1 (1.4)	13.4 (1.0)	13.3 (1.1)
Weight (kg)	Mean (SD)	69.8 (11.2)	69.5 (10.9)	70.2 (11.6)	72.1 (10.7)	71.6 (9.2)	70.8 (11.9)	73.5 (9.5)	73.0 (12.9)
BMI (kg/m^2^)	Mean (SD)	26.1 (2.7)	26.2 (2.8)	26.0 (2.5)	26.6 (2.7)	26.9 (2.6)	26.3 (3.0)	26.7 (2.6)	26.5 (2.7)
Total protein intake (g/kg BW/day)	Median (IQR)	1.0 (0.8,1.2)	1.0 (0.8,1.2)	1.0 (0.9,1.2)	1.1 (0.9,1.5)	0.9 (0.8,1.1)	0.9 (0.8,1.0)	1.5 (1.3,1.7)	1.5 (1.3,1.7)
Total energy intake (kcal/day)	Median (IQR)	1653 (1411,1964)	1612 (1404,1919)	1698 (1423,2028)	1880 (1606,2208)	1907 (1587,2163)	1815 (1603,2011)	1964 (1601,2275)	1856 (1638,2372)
Dietary vitamin D intake (µg/day)	Median (IQR)	2.0 (1.0, 4.0)	1.9 (0.9, 3.7)	2.0 (1.1, 4.1)	2.1 (1.0, 4.3)	2.6 (1.1, 6.5)	1.9 (0.9, 4.1)	2.2 (1.0, 3.8)	2.0 (1.0, 4.8)
Serum calcidiol (nmol/L)	Median (IQR)	48.0 (34.0,66.0)	49.0 (34.0,65.0)	48.0 (33.0,66.0)	64.0 (39.0,80.0)	43.0 (30.0,58.5)	41.0 (28.0,57.0)	81.0 (69.0,96.0)	77.0 (68.0,88.0)
PTH (pmol/L)	Median (IQR)	5.7 (4.2,7.7)	5.7 (4.2,7.5)	5.9 (4.2,8.1)	5.3 (3.8,7.4)	5.8 (4.5,7.7)	6.1 (5.2,8.2)	4.6 (3.6,6.9)	4.3 (3.4,5.7)
Calcium corrected for albumin (mmol/L)	Mean (SD)	2.3 (0.14)	2.31 (0.15)	2.29 (0.12)	2.31 (0.14)	2.30 (0.15)	2.30 (0.17)	2.33 (0.11)	2.33 (0.12)
Creatinine (µmol/L)	Mean (SD)	76.1 (21.7)	76.7 (21.7)	75.5 (21.8)	76.3 (20.3)	76.2 (18.7)	79.7 (21.0)	74.4 (21.3)	74.4 (20.9)
eGFR (mL/min/1.73m^2^)	Mean (SD)	73.5 (15.9)	73.0. (16.4)	74.1 (15.4)	73.9 (15.6)	73.0 (15.4)	71.5 (15.8)	77.1 (14.4)	74.3 (17.2)
ALAT (U/L)	Mean (SD)	20.2 (8.1)	20.1 (8.5)	20.2 (7.6)	23.1 (14.6)	23.4 (17.7)	20.9 (9.0)	25.7 (18.0)	22.3 (8.3)
ASAT (U/L)	Mean (SD)	23.6 (6.4)	23.4 (6.7)	23.9 (6.1)	25.1 (14.5)	26.7 (23.7)	24.2 (6.8)	25.0 (8.0)	23.9 (6.0)
ALP (U/L)	Mean (SD)	77.4 (25.2)	78.3 (27.6)	76.3 (22.3)	78.2 (28.5)	81.5 (40.0)	79.3 (22.5)	77.3 (23.0)	72.8 (18.1)
ɣ-GT (U/L)	Mean (SD)	29.3 (30.9)	29.7 (30)	29.0 (31.9)	30.7 (32.5)	31.8 (28.2)	26.5 (20.5)	36.1 (50.2)	26.9 (16.9)

CT1, former RCT Control group receiving 1 serving of WP-MND during OLE; CT2, former RCT Control group receiving 2 servings of WP-MND during OLE; TT1, former RCT Test group receiving 1 serving of WP-MND during OLE; TT2, former RCT Test group receiving 2 servings of WP-MND during OLE

### Protein intake and kidney function

During the RCT, supplementation with WP-MND led to a significant increase in median total protein intake from 1.0 (IQR: 0.9, 1.2) g/kg BW/day to 1.5 (IQR: 1.3, 1.7) g/kg BW/day in the test group (*p* < 0.001), with no change in the control group (T vs. C: *p* < 0.001). Median protein intake (g/kg BW/day) increased in the former RCT control groups during the OLE study period from 0.9 (IQR: 0.8, 1.1) to 1.3 (IQR: 1.1, 1.5) in the CT1 (*p* < 0.001) and to 1.4 (IQR: 1.3, 1.7) g/kg BW/day in the CT2 group (*p* < 0.001). In the TT1 group that consumed one serving of WP-MND during the OLE study period after consumption of 2 servings during the RCT, median protein intake decreased from 1.5 (IQR: 1.3, 1.7) to 1.2 (IQR: 1.1, 1.4) g/kg BW/day (*p* < 0.001) versus no change in the TT2 group (TT1 vs. TT2: *p* < 0.001).

**Fig. 2:**
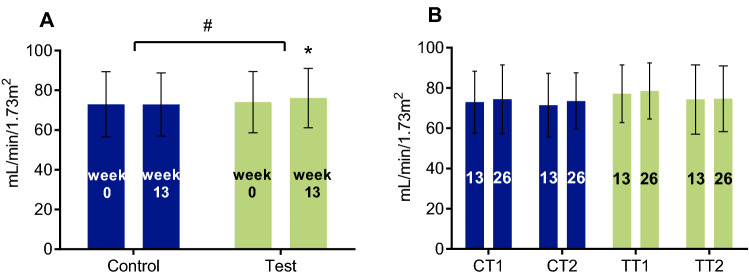
Estimated glomerular filtration rate (eGFR) in the RCT (**a**), and OLE population (**b**). Results are expressed as median (IQR). This figure shows the eGFR in the RCT population at baseline (week 0) and after intervention (week 13) with 2 servings of a vitamin D, calcium and leucine enriched whey protein medical nutrition drink (WP-MND) (Test) or Control product (Control) (**a**), and in the open-label extension (OLE) population at baseline (week 13) and after intervention (week 26) with one or two servings of WP-MND) (**b**). CT1, former RCT Control group receiving 1 serving of WP-MND during OLE; CT2, former RCT Control group receiving 2 servings of WP-MND during OLE; TT1, former RCT Test group receiving 1 serving of WP-MND during OLE; TT2, former RCT Test group receiving 2 servings of WP-MND during OLE. ^#^Significant difference in change between Test and Control groups. * Significant change within group

The study population had a median eGFR of 77 (IQR: 63, 87) mL/min/1.73 m^2^ at baseline, prior to the RCT. 13-week supplementation with 2 servings of the WP-MND during the RCT led to a small increase in eGFR in the test group (median change 1.0 (IQR: − 2.8, 4.2) mL/min/1.73 m^2^; *p* = 0.013) versus no significant change in the control group (median change − 1.1 (IQR: − 4.9, 2.8) mL/min/1.73 m^2^; *p* = 0.067)(T vs. C: *p* = 0.002) (Fig. 2a). No change in eGFR was observed during the OLE part of the study (Fig. 2b). Some of the participants (T: *n* = 36 and C: *n* = 39) had an eGFR between 30 and 60 mL/min/1.73 m^2^ at baseline, before the start of the RCT, which is defined as moderate chronic kidney disease (CKD). In this vulnerable subgroup, the same trend in eGFR was observed as in the total study population. During the RCT, eGFR increased in participants with moderate CKD at baseline in the test group from median 52 (IQR: 48.1, 56.2) to 56.2 (IQR: 46.5, 63.8; *p* = 0.007) mL/min/1.73 m^2^ vs. no change in the control group (T vs. C: NS). No further change was observed during OLE within or between the moderate CKD subgroups.

### Leucine intake

Based on the reported protein intake data and the estimation that dietary protein contains on average 7.8% leucine [27], the total daily leucine intake from the diet and the WP-MND was calculated. The median baseline intake of leucine from the diet at the start of the RCT was estimated to be around 0.078 g/kg BW/day (1.0 g protein/kg BW/day). At week 13, leucine intake from the diet was estimated to be around 0.070 g/kg BW/day (0.9 g protein/kg BW/day) in 50% (median) and 0.086 g/kg BW/day (1.1 g protein/kg BW/day) in 75% (upper IQR) of the test group. The WP-MND supplement contributed to an additional 6 g leucine per day, representing 0.086 g/kg BW/day based on a median bodyweight of 70 kg. In total, leucine intake in the WP-MND group was 0.171 g/kg BW/day in 75% of subjects, which is well below the TUL for leucine of 0.5 g/kg BW/day [28–30].

### Vitamin D and calcium metabolism

**Fig. 3:**
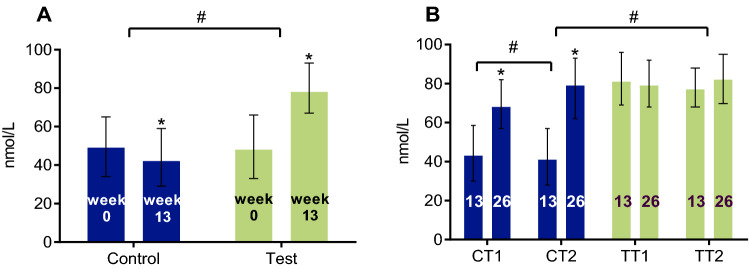
Serum levels of 25-hydroxy-vitamin D (calcidiol) in the RCT (**a**), and OLE population (**b**). Results are expressed as median (IQR). This figure shows the serum concentrations of calcidiol in the RCT population at baseline (week 0) and after intervention (week 13) with 2 servings of a vitamin D, calcium and leucine enriched whey protein medical nutrition drink (WP-MND) (Test) or control product (Control) (**a**), and in the OLE population at baseline (week 13) and after intervention (week 26) with one or two servings of WP-MND (**b**). CT1, former RCT Control group receiving 1 serving of WP-MND during OLE; CT2, former RCT Control group receiving 2 servings of WP-MND during OLE; TT1, former RCT Test group receiving 1 serving of WP-MND during OLE; TT2, former RCT Test group receiving 2 servings of WP-MND during OLE. ^#^Significant difference in change between Test and Control groups. * Significant change within group

After 13 weeks of intervention with 2 servings of the WP-MND in the test group, a significant increase in median serum calcidiol (*p* < 0.001) was observed versus a significant decrease (*p* < 0.001) in the control population (T vs. C: *p* < 0.001) (Fig. 3a). No further increase was observed in either of the former test groups (TT1 and TT2) during the OLE study period (13 vs. 26 weeks) (Fig. 3b). On the other hand, a significant increase in calcidiol levels was observed in the participants who switched from the control product during the RCT to the test product in the OLE study period (both CT1, CT2: *p* < 0.001) and most in the group that received 2 servings of the WP-MND (CT1 vs. CT2: *p* < 0.001).

Fourteen participants had a serum calcidiol concentration ≥ 100 nmol/L at baseline, prior to the RCT (T: *n* = 6; C: *n* = 8). In 20 participants with normal calcidiol levels at baseline, calcidiol increased above 100 nmol/L during the RCT (T: *n* = 18, C: *n* = 2). During the OLE study period, the number of participants with serum calcidiol levels ≥ 100 nmol/L decreased in the former test groups (T: n = 20 → 16) and increased in the former control groups (C: *n* = 6 → 9). In 13 OLE participants (T: *n* = 10; C: *n* = 3) with calcidiol levels ≥ 100 at week 13, serum calcidiol increased further at week 26 (range of increase: 2–38 nmol/L), independent of the number of servings.

**Fig. 4:**
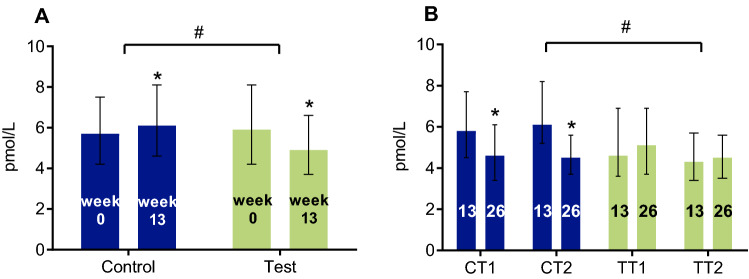
Serum levels of parathyroid hormone (PTH) in the RCT (**a**), and OLE population (**b**). Results are expressed as median (IQR). This figure shows the serum concentrations of PTH in the RCT population at baseline (week 0) and after intervention (week 13) with 2 servings of a vitamin D, calcium and leucine enriched whey protein medical nutrition drink (WP-MND) (Test) or Control product (Control) (**a**), and in the OLE population at baseline (week 13) and after intervention (week 26) with one or two servings of WP-MND (**b**). CT1, former RCT Control group receiving 1 serving of WP-MND during OLE; CT2, former RCT Control group receiving 2 servings of WP-MND during OLE; TT1, former RCT Test group receiving 1 serving of WP-MND during OLE; TT2, former RCT Test group receiving 2 servings of WP-MND during OLE. ^#^Significant difference in change between Test and Control groups. * Significant change within group

During the RCT, serum levels of PTH decreased in the test group (*p* < 0.001) and increased in the control group (*p* = 0.006) (Fig. 4a). Serum levels of PTH did not change with continued supplementation of a WP-MND but decreased significantly in participants who switched from control to test product during the OLE study period (*p* < 0.001) (Fig. 4b).

**Fig. 5:**
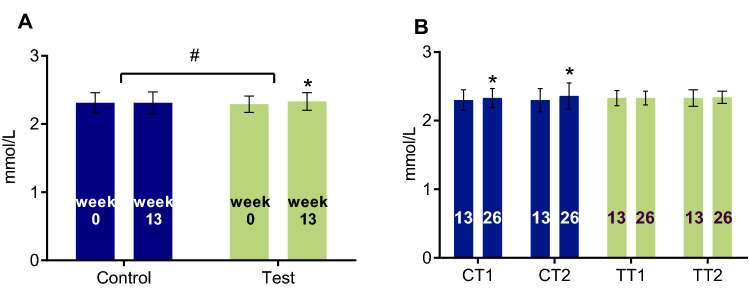
Albumin-adjusted serum levels of calcium in the RCT (**a**), and OLE population (**b**). Results are expressed as mean (SD). This figure shows the albumin-adjusted serum concentrations of calcium in the RCT population at baseline (week 0) and after intervention (week 13) with 2 servings of a vitamin D, calcium and leucine enriched whey protein medical nutrition drink (WP-MND) (Test) or Control product (Control) (**a**), and in the OLE population at baseline (week 13) and after intervention (week 26) with one or two servings of WP-MND (**b**). CT1, former RCT Control group receiving 1 serving of WP-MND during OLE; CT2, former RCT Control group receiving 2 servings of WP-MND during OLE; TT1, former RCT Test group receiving 1 serving of WP-MND during OLE; TT2, former RCT Test group receiving 2 servings of WP-MND during OLE. ^#^Significant difference in change between Test and Control groups. * Significant change within group

During the RCT, the albumin-adjusted serum calcium concentration increased in the test group from 2.29 to 2.33 mmol/L (*p* < 0.001) versus no change in the control group (mean: 2.31 mmol/L; T vs. C: *p* < 0.001) (Fig. 5a). During the OLE study period, calcium levels increased in the former control population (CT1: *p* = 0.017; CT2: *p* = 0.003) whereas no further increase was observed in the former test population (Fig. 5b). Although albumin-corrected calcium levels > 2.55 mmol/L were observed, the proportion of participants with hypercalcaemia was not different between groups (Table 2). At baseline, 12 subjects already manifested albumin-corrected calcium levels above 2.55 mmol/L (T: *n* = 3; C: *n* = 9). During the RCT, a similar number of subjects in the test and control group developed hypercalcaemia (T: *n* = 3; C: *n* = 3), while 8 (T: *n* = 2; C: *n* = 6) of the 14 subjects with hypercalcemia at week 13 already had elevated levels at baseline. This suggests no direct association between the development of hypercalcemia and supplementation of WP-MND but may point to a surprisingly higher prevalence of hypercalcemia in this sarcopenic older population. In OLE extension with 2 servings per day (CT2), 2 subjects maintained the elevated calcium levels and 3 new subjects were found with calcium levels above 2.55 mmol/L. In the OLE group taking 1 serving a day (CT1), 3 subjects maintained higher calcium levels, while 2 subjects with the elevation of calcium above 2.55 mmol/L were found. The presence of hypercalcemia at baseline or development of hypercalcemia during the study in any of the groups showed no connection to evolvement over time of PTH, calcidiol, creatinine, and eGFR.

**Table 2:** Prevalence (*n*, %) of hypercalcaemia (albumin corrected calcium > 2.55 mmol/L) during RCT and open-label extension of the study (OLE)

	Control	Test	CT1	CT2	TT1	TT2
Baseline RCT (week 0)	9 (4.8%)	3 (1.7%)	3 (4.3%)	4 (7.7%)	2 (3.4%)	0 (0.0%)
End RCT/baseline OLE (week 13)	9 (5.8%)	4 (2.9%)	4 (5.6%)	2 (3.5%)	1 (1.7%)	1 (2.4%)
End OLE (week 26)	10 (8.7%)	1 (1.1%)	5 (6.9%)	5 (11.6%)	1 (1.8%)	0 (0.0%)

CT1, former RCT Control group receiving 1 serving of WP-MND during OLE; CT2, former RCT Control group receiving 2 servings of WP-MND during OLE; TT1, former RCT Test group receiving 1 serving of WP-MND during OLE; TT2, former RCT Test group receiving 2 servings of WP-MND during OLE

At the start of RCT, none of the 14 participants with calcidiol levels above 100 nmol/L had albumin-corrected calcium levels above 2.55 mmol/L. At week 13 (end RCT and start OLE), 3 out of 26 participants with calcidiol above 100 nmol/L (from the former test group) had albumin-corrected calcium levels above 2.55 mmol/L. At week 26 (end OLE), 3 out of 25 participants had both levels elevated (1 from the TT1, 1 from the TT2 and 1 from the CT2 group) of which for one of them (from the TT2 group) this was already the case at baseline OLE.

### General safety, vital signs

Parameters of liver function did not increase during the RCT or OLE study period and values were within the range of reference values (Table 1).

At baseline, heart rate was significantly higher in the test group compared to the control group (*p* = 0.011), but no significant changes in heart rate, systolic and diastolic blood pressure were observed between the two groups after 13 weeks of intervention (Table 3 in Appendix). At OLE baseline (week 13), heart rate in TT1 was significantly lower compared to the TT2 group (*p* = 0.018), but no other significant differences were found between the groups at week 26. Similarly, no significant differences were found in systolic or diastolic blood pressure between the different groups (CT1 vs. CT2; TT1 vs. TT2; CT2 vs. TT2) during the OLE extension (Table 4 in Appendix).

### Adverse events

During the RCT, a total of 557 adverse events were reported among 240 subjects. From those, 193 were assessed as being related to the study products, without a significant difference between the test group (46 subjects; 25.0%) and the control group (54 subjects; 27.7%) (*p* = 0.562). In the OLE study period, a total of 216 adverse events were reported among 112 subjects. From those, 57 were assessed as being related to the study product. The number of subjects reporting one or more related adverse events was significantly higher in the TT2 group (9 subjects; 20.9%) compared to the TT1 group (only 3 subjects; 5%) (*p* = 0.026). Since other between-groups comparisons did not show a significant difference (TT2 vs. CT2 and CT2 vs. CT1), there was no apparent dose-relationship and the data did not point to any concern. The most commonly reported related adverse events were gastro-intestinal (such as abdominal pain and nausea) or metabolic and nutritional disorders (such as hyperglycaemia). No treatment-related serious adverse events occurred during the RCT and OLE study periods. Moreover, no related adverse (serious or non-serious) events, including nephrolithiasis (kidney stones), were observed in participants with serum calcidiol levels ≥ 100 nmol/L or with albumin-adjusted calcium levels > 2.55 mmol/L, at any of the time points.

### Gastrointestinal tolerability

The incidence and the severity of the self-reported GI symptoms (nausea, belching, feeling of fullness, vomiting, abdominal distension, flatulence, diarrhoea, constipation, dry mouth and thirst) did not differ between the control and the test group at baseline or the end of the RCT (Table 5 in Appendix). Moreover, no difference was observed between the two groups with regards to the changes at week 13 relative to the baseline for any of the GI symptoms. The OLE study also showed no difference in the incidence or severity of the GI symptoms between the groups (CT1 vs. CT2; TT1 vs. TT2; CT2 vs. TT2), except for diarrhoea which was significantly lower in CT2 compared to CT1 (*p* = 0.01) (Table 6 in Appendix).

## Discussion

6-month intervention with up to 2 servings of this WP-MND in addition to a regular diet neither impaired kidney function nor caused vitamin D and/or calcium toxicity and is not related to impaired liver function and vital signs, increased gastro-intestinal intolerance or -adverse events.

### Protein intake and kidney function

The consistent high protein intake in line with recommendations, especially in participants receiving 2 servings of WP-MND throughout the entire 26-week study period (during RCT and OLE), enabled evaluation of the effect of a prolonged high protein intake on kidney function.

Concerns regarding the adverse effect of a high protein diet on kidney function are related to glomerular hyperfiltration and hypertensive effects [31–33]. During the RCT, eGFR increased in the test vs. no change in the control group (*p* = 0.002). The same trend was observed in the subgroup of participants with moderate CKD at baseline. During OLE, no (further) change in eGFR was observed in any of the (sub)groups. The alteration of eGFR in response to a high protein diet is in line with observations by others and is thought to be an adaptive response to the protein feeding and not the development of CKD per se [31, 33, 34]. The observed plateau in eGFR after 13 weeks of treatment with 2 servings of WP-MND during the OLE period, supports this notion of adaptation. Moreover, the observed increase and subsequent plateau of eGFR after 13 weeks of treatment in older people with moderate CKD suggest that even those individuals still tolerate a high protein diet. However, caution is still warranted, and kidney function should be monitored on a regular basis in people on a high protein diet with moderate CKD, as recommended [7, 9, 12]. Furthermore, blood pressure did not change over time and was not different between groups, which supports the assumption that the observed increase of eGFR was not an indication of early kidney failure.

### Vitamin D and calcium metabolism

Calcidiol levels increased with the intake of 2 servings of WP-MND, providing 40 µg vitamin D, and reached a plateau at ~ 80 nmol/L after 3 months of supplementation. Moreover, 1 serving of WP-MND providing 20 µg vitamin D also increased calcidiol levels after 3 months, although to a lesser extent (~ 70 nmol/L), hence suggesting a dose–response effect. When considering a baseline diet with a median vitamin D intake of 2.0 (IQR: 1.0, 4.0) µg/day at the beginning of the RCT and 2.1 (IQR: 1.0, 4.3) µg/day at the beginning of OLE (Table 1), the total intake of vitamin D did not exceed 50 µg/day. This amount is well below the TUL for vitamin D intake at 100 µg per day set by EFSA [35]. For adults, EFSA selected hypercalcemia as the indicator of vitamin D toxicity that may occur when plasma levels of calcidiol increase above 100 nmol/L [5, 15, 36–39]. In our study, some participants had calcidiol levels ≥ 100 nmol/L with or without hypercalcemia (albumin-corrected calcium levels > 2.55 mmol/L) at baseline or during the study. However, no causal relation was observed between high serum calcidiol and calcium levels and no related adverse events were reported. An explanation for high plasma calcidiol levels may be an enhanced endogenous skin vitamin D production following exposure to sunlight [40, 41] or clandestine consumption of vitamin D containing food supplements.

Although no relationship was observed between hypercalcidiol and hypercalcemia, the overall increase in serum calcium was significant but marginal in the test group during the RCT, and in the control group during OLE. This increase was observed to plateau after 3 months of supplementation with 2 servings of WP-MND. The total group average serum calcium level at baseline was 2.30 mmol/L (not different between groups), which is within the reference range of 2.15–2.6 mmol/L [24]. Twelve participants had hypercalcemia at baseline of the RCT, which is a surprisingly higher prevalence than reported in other populations, such as in the community and hospital (0.2–4% [42, 43]). This warrants confirmation of potentially unknown hypercalcemia cases among the sarcopenic population in future research. The prevalence of hypercalcemia did not change during the course of the RCT and the OLE study periods, with some participants maintaining hypercalcemia and others developing hypercalcemia, independent of the treatment group. The presence or development of hypercalcemia could not be linked to clinically relevant safety indicators or adverse events. As expected, serum parathyroid hormone (PTH) levels decreased with the intake of the WP-MND. Therefore, hyperparathyroidism was unlikely to be the cause of observed hypercalcemia. Another cause for hypercalcemia could be due to the calcium provided (1000 mg in 2 servings of WP-MND on top of the diet providing on average about 1000 g/day of calcium), leaving a margin of 500 mg before exceeding the TUL as established by EFSA [44]. However, the Panel considers that no relationship has been established between long-term calcium intakes at 2.5 g/day from diet and supplements and increased risk of nephrolithiasis.

### General safety and gastrointestinal tolerability

There was no difference in the number and origin of the adverse events between test and (former) control groups. Most of the adverse events were gastrointestinal, but the incidence and severity of gastrointestinal symptoms did not differ between the treatment groups. No potentially treatment-related serious adverse events were observed. Moreover, no adverse (serious or non-serious) events were observed in participants with serum calcidiol levels ≥ 100 nmol/L or with albumin-adjusted calcium levels > 2.55 mmol/L, at any of the time points. Finally, vital signs did not change throughout the study, and parameters of liver function were within the reference range and did not increase in any of the groups during the RCT or OLE study period.

### Strengths and limitations

The main strength of this investigation is the evaluation of the safety of an effective medical nutrition drink in a study group of sarcopenic older adults (*n* = 380) [19]. Detailed information was collected about the impact of 1–2 servings of WP-MND on protein intake, kidney function, vitamin D and calcium metabolism. Furthermore, participants with moderate CKD, supra-normal levels of calcidiol or hypercalcemia were separately analyzed and scrutinized for adverse effects. Another strength is that we observed a plateau after 3-months of WP-MND supplementation for eGFR and plasma levels of calcidiol, calcium and PTH, suggesting that the study period was likely long enough to demonstrate that consumption of two servings of WP-MND is safe in the longer term. A study limitation may be that treatment groups were small during OLE, although observed effects point in the same direction as during the RCT. Another limitation may be that we did not determine plasma ammonia and insulin resistance over time, which are indicative of short-term leucine toxicity and potential negative effects of leucine supplementation in longer term [45]. Furthermore, eGFR should perhaps have been monitored over an even longer period to ensure that the observed increase in eGFR in the test population just represented an adaptation to the high protein intake.

In conclusion, a 6-month intake of a vitamin D, calcium and leucine-enriched whey protein medical nutrition drink in addition to a regular diet does not impair kidney function or disturb vitamin D and calcium metabolism in sarcopenic older adults. Furthermore, WP-MND does not affect liver function and vital signs and is well tolerated by the gastrointestinal tract. In line with the protein recommendations, we do recommend monitoring of kidney function in consumers with moderate CKD [9]. For kidney protection in general, sufficient fluid intake is recommended for proper hydration [46].

## Appendix

See Tables 3, 4, 5, 6.

**Table 3:** Measured vital signs at baseline of the RCT study (week 0) and at the end of the RCT study (week 13) in the control and the test group

Vital signs	Statistic	Week 0 (baseline RCT)		Week 13 (end RCT, baseline OLE)	
		Control (*n* = 195)	Test (*n* = 184)	Control (*n* = 196)	Test (*n* = 184)
Heart rate (bpm)	Mean (SD)	69.2 (11.3)	72.1 (11.1)*	69.5 (11.2)	71.5 (10.3)
Systolic blood pressure (mmHg)	Mean (SD)	141 (20.8)	137 (18.2)	138.8 (20.4)	134.8 (15.8)
Diastolic blood pressure (mmHg)	Mean (SD)	76 (10.4)	75.6 (10.3)	76.4 (10.2)	74.7 (9.5)

CT1, former RCT Control group receiving 1 serving of WP−MND during OLE; CT2, former RCT Control group receiving 2 servings of WP−MND during OLE; TT1, former RCT Test group receiving 1 serving of WP−MND during OLE; TT2, former RCT Test group receiving 2 servings of WP−MND during OLE

*Significant difference between control and test; *p *value = 0.011 (*t* test)

**Table 4:** Vital signs at baseline of the open-label extension (week 13) and at the end of the OLE study period (week 26)

Vital signs	Statistic	Week 13 (baseline OLE)				Week 26			
		RCT control (*n* = 130)		RCT tests (*n* = 103)		RCT control (*n* = 130)		RCT tests (*n* = 103)	
		CT1 (*n* = 73)	CT2 (*n* = 57)	TT1 (*n* = 60)	TT2 (*n* = 43)	CT1 (*n* = 73)	CT2 (*n* = 57)	TT1 (*n* = 60)	TT2 (*n* = 43)
Heart rate (bpm)	Mean (SD)	67.1 (10.9)	71 (11.3)	69.2 (11.5)	74.4 (9.7)*	67.8 (10.8)	69.6 (10.4)	70.4 (10.3)	73.4 (9.6)
Systolic blood pressure (mmHg)	Mean (SD)	142 (22.2)	135.5 (19)	135 (16.5)	134.1 (16.8)	138.1 (19)	132.5 (17.3)	138.2 (16.7)	134.5 (14.4)
Diastolic blood pressure (mmHg)	Mean (SD)	76.9 (10.1)	75.2 (9.1)	76.2 (10.5)	72.5 (8.8)	73.9 (11)	74 (9.7)	76 (9.1)	74.1 (9.4)

CT1, former RCT Control group receiving 1 serving of WP-MND during OLE; CT2, former RCT Control group receiving 2 servings of WP-MND during OLE; TT1, former RCT Test group receiving 1 serving of WP-MND during OLE; TT2, former RCT Test group receiving 2 servings of WP-MND during OLE

^*^Significant difference between TT1 and TT2; *p *value = 0.018 (*t* test)

**Table 5:** Incidence of the gastrointestinal symptoms at baseline of the RCT study (week 0) and at the end of the RCT (week 13) in the test and the control group

GI symptoms	Statistic	Week 0 (baseline RCT)		Week 13 (end RCT, baseline OLE)	
		Control (*n* = 195)	Test (*n* = 184)	Control (*n* = 195)	Test (*n* = 184)
Nausea	*n* (%)	21 (10.8%)	14 (7.6%)	15 (9.5%)	17 (11.9%)
Belching	*n* (%)	48 (24.6%)	44 (23.9%)	36 (22.8%)	36 (25.2%)
Feeling of fullness	*n* (%)	44 (22.6%)	39 (21.2%)	49 (31.0%)	48 (33.6%)
Vomiting	*n* (%)	4 (2.1%)	4 (2.2%)	3 (1.9%)	1 (0.7%)
Abdominal distension	*n* (%)	46 (23.6%)	45 (24.5%)	33 (20.9%)	29 (20.3%)
Flatulence	*n* (%)	78 (40%)	82 (44.6%)	46 (29.1%)	54 (37.8%)
Diarrhoea	*n *(%)	20 (10.3%)	19 (10.3%)	20 (12.7%)	15 (10.6%)
Constipation	*n* (%)	48 (24.6%)	60 (32.6%)	28 (17.7%)	36 (25.2%)
Dry mouth	*n* (%)	71 (36.9%)	80 (43.5%)	53 (33.5%)	53 (37.1%)
Thirst	*n* (%)	34 (17.4%)	39 (21.2%)	29 (18.4%)	40 (28%)

CT1, former RCT Control group receiving 1 serving of WP-MND during OLE; CT2, former RCT Control group receiving 2 servings of WP-MND during OLE; TT1, former RCT Test group receiving 1 serving of WP-MND during OLE; TT2, former RCT Test group receiving 2 servings of WP-MND during OLE

**Table 6:** Incidence of the gastrointestinal symptoms at baseline of the open-label extension (week 13) and at the end of OLE study period (week 26)

GI symptoms	Statistic	Week 13 (baseline OLE)				Week 26				
		RCT control (*n* = 130)		RCT test (*n* = 103)		RCT Control (*n* = 130)			RCT Test (*n* = 103)	
		CT1 (*n* = 73)	CT2 (*n* = 57)	TT1 (*n* = 60)	TT2 (*n* = 43)	CT1 (*n* = 73)		CT2 (*n* = 57)	TT1 (*n* = 60)	TT2 (*n* = 43)
Nausea	*n* (%)	5 (6.8%)	6 (10.5%)	4 (6.7%)	4 (9.3%)	7 (9.7%)		5 (10.9%)	7 (12.5%)	4 (10.3%)
Belching	*n* (%)	17 (23.3%)	12 (21.1%)	16 (26.7%)	13 (30.2%)	13 (18.1%)		12 (26.1%)	15 (26.8%)	9 (23.1%)
Feeling of fullness	*n* (%)	22 (30.1%)	16 (28.1%)	18 (30%)	16 (37.2%)	21 (29.2%)		19 (41.3%)	15 (26.8%)	13 (33.3%)
Vomiting	*n* (%)	0 (0%)	2 (3.5%)	0 (0%)	0 (0%)	2 (2.8%)		1 (2.2%)	2 (3.6%)	1 (2.6%)
Abdominal distension	*n* (%)	12 (16.4%)	12 (21.1%)	13 (21.7%)	4 (9.3%)	15 (20.8%)		7 (15.2%)	12 (21.4%)	8 (20.5%)
Flatulence	*n* (%)	19 (26%)	17 (29.8%)	22 (36.7%)	13 (30.2%)	25 (34.7%)		15 (32.6%)	22 (39.3%)	9 (23.1%)
Diarrhoea	*n* (%)	8 (11%)	7 (12.3%)	6 (10.2%)	6 (14%)	12 (16.7%)		3 (6.5%)*	5 (8.9%)	4 (10.3%)
Constipation	*n* (%)	12 (16.4%)	9 (15.8%)	17 (28.3%)	8 (18.6%)	13 (18.1%)		13 (28.3%)	14 (25%)	7 (17.9%)
Dry mouth	*n* (%)	24 (32.9%)	22 (38.6%)	19 (31.7%)	18 (41.9%)	25 (34.7%)		16 (34.8%)	19 (33.9%)	14 (35.9%)
Thirst	*n* (%)	11 (15.1%)	9 (15.8%)	17 (28.3%)	10 (23.3%)	20 (27.8%)		9 (19.6%)	13 (23.2%)	11 (28.2%)

CT1, former RCT Control group receiving 1 serving of WP-MND during OLE; CT2, former RCT Control group receiving 2 servings of WP-MND during OLE; TT1, former RCT Test group receiving 1 serving of WP-MND during OLE; TT2, former RCT Test group receiving 2 servings of WP-MND during OLE.

^*^Significant difference between CT1 and CT2; *p* value = 0.01 (Mann–Whitney test)
